# Maturation and Patency Rates in Basilic Transposition Arteriovenous Fistula Under Regional Versus General Anesthesia: A Single-Center, Retrospective, Observational Study

**DOI:** 10.7759/cureus.16991

**Published:** 2021-08-08

**Authors:** Waryam Muhammad Saleh, Zia U Rehman, Shiraz Hashmi

**Affiliations:** 1 Section of Vascular Surgery, Department of Surgery, Aga Khan University Hospital, Karachi, PAK; 2 Department of Vascular and Endovascular Surgery, Shaheed Mohtarma Benazir Bhutto Institute of Trauma, Karachi, PAK; 3 Department of Surgery, Aga Khan University Hospital, Karachi, PAK

**Keywords:** basilic transposition arteriovenous fistula, regional anesthesia, maturation, patency, complication rates

## Abstract

Background

Basilic transposition arteriovenous fistula (BT AVF) is a viable option for dialysis-dependent patients, which can be performed under either general or regional anesthesia. Regional anesthesia is reported to cause vascular dilatation during the perioperative period, leading to improved fistula success. Regional anesthesia is also considered safe as compared to general anesthesia in terms of hemodynamic stability. Limited and conflicting data are available comparing regional versus general anesthesia in terms of fistula maturation and patency. We aimed to compare the maturation, one-year patency rates, and complication rates in patients undergoing single-stage BT AVF in regional versus general anesthesia.

Methods

This retrospective observational study was conducted on patients undergoing single-stage BT AVF from January 2016 to December 2019. Patients were divided into regional (RA) vs. general anesthesia (GA) groups and compared in terms of maturation, one-year patency, and perioperative complication rates.

Results

Out of 152 patients, 110 (72.37%) were in GA while 42 (27.63%) were in the RA group. Elderly, female, diabetic, ischemic heart disease, and American Society of Anesthesiologists (ASA) class IV patients were more in the RA group. Other comorbid and vascular access-related factors were comparable between the groups. A trend toward higher maturation rates (97.6% vs. 92.1%) and one-year patency rates (62.5% vs. 56.6%) was observed in the RA vs. GA group, however, the difference did not attain statistical significance, p=0.359 and p=0.327, respectively. The rate of access abandonment was higher in the GA group (43.4% vs. 37.5%). The most prevalent cause of abandonment was death in the RA group while it was access failure in the GA group. Overall complication rates were comparable between both groups (20.2 % vs. 17.5%, p=0.816).

Conclusion

Regional anesthesia is a useful technique with potentially improved maturation and patency rates. Nevertheless, an assumed benefit of regional anesthesia in terms of anesthesia-related complications was not observed.

## Introduction

Chronic kidney disease (CKD) is a debilitating disease and significant public health problem, affecting 12% - 18.7% of the population in Europe as well as Asia [1-2]. The prevalence of CKD is as high as 23.3% in Pakistan [3]. Patients with end-stage renal disease (ESRD) need renal replacement therapy in form of renal transplant or dialysis. A significant proportion of these patients cannot undergo a renal transplant and need lifelong dialysis. Good quality, durable angioaccess is a major contributing factor in determining survival in this sub-set of the CKD population. An arteriovenous fistula (AVF) is considered the best vascular access because of longer patency rates and lower intervention and complication rates [4-7]. Radiocephalic AVF (RC AVF) and brachiocephalic AVF (BC AVF) are considered primary and preferred autologous accesses. A basilic transposition arteriovenous fistula (BT AVF), first described in 1976 by Dagher et al., has been increasingly accepted as a viable option for secondary vascular access [8] and it is indicated when adequate superficial veins in the forearm or arm are not available for primary access procedures. It has higher venous flow with high patency and maturation rates but BT AVF needs longer operative time and longer dissection of basilic vein for its superficialization and transposition, requiring either general (GA) or regional anesthesia (RA). BT AVF is performed either as single-stage if the vein is good-sized, but if it is of smaller size, initially only brachio-basilic AVF is formed in the first stage and superficialization of AVF is done at the second stage, four to six weeks after the initial procedure [9].

In general anesthesia (GA) patients are made unconscious with or without muscle relaxation with the placement of an endotracheal tube or laryngeal mask airway (LMA) while in RA local anesthetic agent is infiltrated around nerves, thus making the area supplied by these nerves numb. For BT AVF RA is administered through a supraclavicular or infraclavicular brachial plexus blockage, usually under ultrasound guidance. ESRD patients are known to be at increased risk of perioperative and postoperative complications [10]. GA is considered riskier than RA mainly because of stress induction that eventually causes hypotension leading to decreased blood flow and thrombosis of fistula [11]. On the contrary, RA is a good alternative, as it avoids hemodynamic instability and stress response compared to GA and causes venous and arterial dilatation by its sympathectomy effect [12]. This vasodilatory effect of RA results in increased venous diameter and peripheral flow intraoperatively and several hours after surgery, thus avoiding perioperative vascular spasm and possibly decreased early access failure and improved maturation and patency rates [13-14]. However, no consensus exists over the optimal mode of anesthesia for AVF creation and it is not clear whether this vasodilatory effect translates into favorable outcomes in terms of improved maturation and patency rates. Although evidence suggests improved patency and maturation and decreased early failure rates when RA is compared with LA [15], data are scarce on the comparative outcomes of RA versus GA, especially on BT AVF formation.

We hypothesize that due to the vasodilatory effect, RA may result in improved outcomes of AVF compared to GA patients undergoing BT AVF. A retrospective, observational study was designed to compare maturation and patency rates at one year as the primary outcome and perioperative complications as the secondary outcome in patients undergoing single-stage BVT AVF under RA or GA.

## Materials and methods

This is a retrospective study conducted on adult patients (aged 18 years or above), undergoing single-stage BT AVF under GA or RA, at the Section of Vascular Surgery, Department of Surgery, Aga Khan University Hospital, Karachi, Pakistan, from January 2016 to December 2018. Patients were recruited by the consecutive, non-probability sampling technique. The cohort was divided into the GA and RA groups. Both groups were followed postoperatively till one year from index surgery for maturation, patency rates at one year, and complication rates. Patients who underwent two-stage brachiobasilic AVF, with missing data or loss of follow-up, underwent BT AVF surgery under local anesthesia with or without sedation were excluded from the study analysis.

Data were collected after getting approval from the institutional ethical review committee (ERC# 2020-5601-14962). The data were extracted from charts and clinical notes on a prestructured proforma.

Information on demographics, including age, gender, and body mass index (BMI), comorbid conditions, and vascular risk factors, such as diabetes, hypertension, hyperlipidemia, ischemic heart disease, tobacco use, and American society of anesthetists (ASA) class, were recorded from clinic notes, anesthesia preoperative assessment forms, and admission notes. Factors affecting fistula maturation and patency such as cause of renal failure, dialysis dependency, previous history of access surgery, previous central cannulation, and size of the basilic vein and brachial artery were also recorded. Data on type of anesthesia (RA or GA), the success of RA, and any anesthesia-related cardiac, respiratory, neurological complications were extracted from the intraoperative anesthesia monitoring form, postoperative notes, follow-up notes in the ward, anesthesia block forms, and discharge summary. Wound-related complications, including infection, dehiscence, bleeding, and hematoma, were recorded from first postoperative follow-up notes, and data on AVF maturation was recorded from clinic notes on six to eight-week follow-up, dialysis unit charts, and subsequent follow-ups. Any unplanned readmissions, AVF related complications, interventions to salvage fistula, and patency rates recorded until one-year follow-up. In cases of fistula abandonment, the cause of abandonment, including death, transplant, and access failure, and the causes of access failure were also explored. Maturation time was defined as time duration (in weeks) from the creation of the fistula to the day the fistula was palpable with discernible margins and good thrill and used successfully with two needles providing prescribed dialysis for more than two-thirds of dialysis sessions within four consecutive weeks [16] while patency was defined as the time period from the creation of fistula till the time it’s blocked or abandoned [16].

Data were analyzed on STATA version 14.2 (StataCorp, College Station, TX). Descriptives of both groups were compared by the independent T-test or Mann Whitney U test for continuous variables and the chi-square or Fisher exact test for categorical variables where appropriate. Kaplan-Meier survival analysis and the log-rank test were used to compare patency rates. A p-value of <0.05 is considered statistically significant.

## Results

One-hundred fifty-two (152) patients who underwent single-stage BT AVF were included in the analysis. One-hundred ten (110; 72.37%) patients underwent the procedure under GA while 42 (27.63%) patients were operated under RA. Thirty-five (35; 83.33%) patients had successful RA. Patients’ basic demographics and clinical factors are shown in Table 1. The female gender was more common in the overall study population. Patients in RA were elderly as compared to GA with a mean age of 59.8 ± 11.2 vs. 53.3 ± 14.6 (p=0.010). The RA group patients had a higher proportion of diabetes (DM), ischemic heart disease (IHD), and American Society of Anesthesiologists (ASA) class IV compared to the GA group. Other comorbids were comparable between the groups.

**Table 1:** Baseline demographics and clinical characteristics of the study population (n=152) CVA: cerebrovascular accident; ASA: American Society of Anesthetists

Variable	Anesthesia		P-value
	General n=110	Regional n=42	
Age (years) ±SD	53.3 ± 14.6	59.8 ± 11.2	0.010
BMI (kg/m^2^) ±SD	26.6 ± 6.5	26.9 ± 4.9	0.802
Gender			
Male n (%)	45 (40.9)	15 (35.7)	0.584
Female n (%)	65 (59.1)	27 (64.3)	
Comorbids			
-Diabetes n (%)	66 (60.0)	33 (78.6)	0.037
-Duration of Diabetes ( years)	18.92 ± 7.82	18.79 ± 7.82	0.944
-Hypertension n (%)	100 (90.9)	41 (97.6)	0.291
-Ischemic Heart Disease n (%)	26 (23.6)	25 (59.5)	<0.001
-CVA n (%)	10 (9.1)	4 (9.5)	0.934
-Tobacco User n (%)	12 (10.9)	4 (9.5)	0.803
ASA Class			
Class III n (%)	104 (94.5)	33 (78.6)	0.003
Class IV n (%)	6 (5.5)	9 (21.4)	
Primary Renal Pathology			
Nephroangiosclerosis	26 (23.6)	9 (21.4)	0.187
Adult Polycystic Kidney Disease	4 (3.6)	0 (0)	
Diabetes Mellitus	59 (53.6)	31 (73.8)	
Obstructive Nephropathy or Lithiasis	5 (4.5)	1 (2.4)	
Malformative Pathologies	1 (0.9)	0 (0)	
Unknown Origin	11 (10)	0 (0)	
Dialysis Dependent	72 (65.45)	25 (59.52)	0.310
Previous Access Surgery	68 (61.8)	22 (52.4)	0.290
Side of Previous Access			
Ipsilateral	35 (51.5)	12 (54.5)	0.960
Contralateral	19 (27.9)	6 (27.3)	
Multiple Access Surgeries	14 (20.6)	4 (18.2)	
Previous Central-Line Cannulation	73 (66.4)	31 (73.8)	0.438
Central cannulation type			
-Bilateral / Ipsilateral	38 (52.1)	13 (41.9)	0.564
-Contralateral	34 (46.6)	17 (54.8)	
-Femoral	1 (1.4)	1 (3.2)	
Basilic Vein Diameter (mm) ±SD	3.2 ± 0.29	3.2 ± 0.19	0.115
Brachial Artery Diameter (mm) ±SD	3.5 ± 0.69	3.7 ± 0.78	0.782
Operative Time (minutes)	151 ± 40.6	140.3 ± 28	0.122

There was no significant difference between groups in terms of dialysis dependency, previous access surgeries, previous history of central and ipsilateral central cannulation, or the size of the basilic vein and brachial artery.

**Figure 1:**
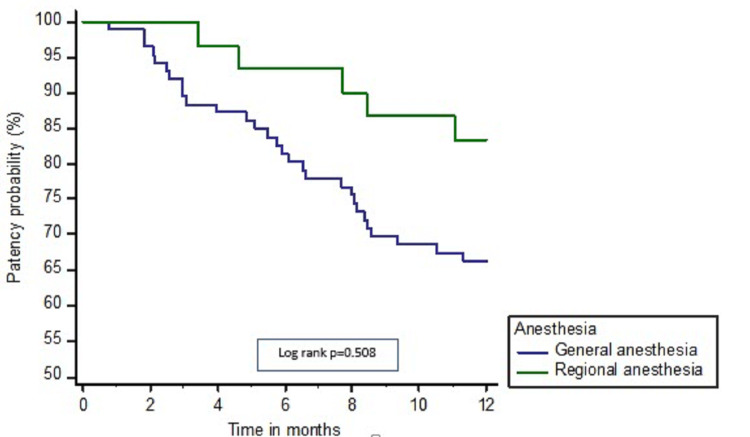
Kaplan Meier’s plots showing a comparison of the patency rates of AVF over 12 months in the GA vs. RA groups AVF: arteriovenous fistula; GA: general anesthesia; RA: regional anesthesia

Outcome variables are given in Table 2. Median follow-up duration was comparable in both the groups; 8.3 (IQR) (7.6, 9.1) vs. 8.0 (7.5, 8.8) months, p=0.852. There is trend toward higher maturation rates (97.6% vs. 92.1%, p=0.186), patency rates at 6 (85.0% vs. 76.8%, p=0.359), and one year (62.5% vs. 56.6%, p=0.327) in the RA vs. GA groups (Table 2). Kaplan-Meier curves endorse the same trends of lower failure and higher patency in the RA group as compared to the GA; however, the log-rank p-value was not significant; p=0.508 (Figure 1).

**Table 2:** Comparison of BT AVF outcomes among general vs. regional anesthesia, n=152 *All were pulmonary complications in both groups. ‡Postoperative length of stay BT AVF: basilic transposition arteriovenous fistula; HDU: high dependency unit

Variable	Anesthesia		P-value
	General n=110	Regional n=42	
Maturation			
Matured	93 (92.1)	40 (97.6)	0.186
Failed	8 (7.9)	1 (2.4)	
Patency at 6 months			
-Patent	76 (76.8)	34 (85.0)	0.359
Abandoned	23 (23.2)	6 (15.0)	
Patency at 1 year			
Patent	56 (56.6)	25 (62.5)	0.327
Abandoned	43 (43.4)	15 (37.5)	
Cause of abandonment			0.017
Death	13 (30.2)	10 (66.7)	
Access failure	30 (69.8)	5 (33.3)	
Transplant	0 (0)	0 (0)	
Cause of access failure			0.739
Thrombosis	23 (76.7)	4 (80)	
Poor flow	6 (20)	1 (20)	
Aneurysm	1 (3.3)	0 (0)	
AVF complications	20 (20.2)	7 (17.5)	0.816
AVF complications type			
Stenosis	15 (75)	7 (100)	
Steal syndrome	3 (15)	0 (0.0)	
Bleeding/Hematoma formation	2 (10)	0 (0.0)	
Intervention	18 (18.2)	5 (12.8)	0.448
Type of Intervention			
Revision of anastomosis	5 (27.8)	0 (0.0)	0.138
Thrombectomy +/- Revision	1 (5.6)	2 (40.0)	
Venoplasty	10 (55.6)	3 (60.0)	
Others	2 (11.1)	0 (0.0)	
Fistula salvaged after intervention	11 (61.1)	4 (80)	0.443
Complications of anesthesia*	1 (0.9)	3 (7.1)	0.032
Length of stay median days (IQR)‡	1 (0-4)	1 (1-9)	0.845
HDU stay	6 (5.5)	4 (9.5)	0.739
ICU stay	0 (00)	2 (4.8)	0.075
Median follow-up time (months) (IQR)	8.3 (7.6, 9.1)	8.0 (7.5, 8.8)	0.852

The rate of abandonment was higher in GA compared to the RA group (43.4% vs. 37.5%). The difference between the reasons for abandonment was statistically significant in the two groups. Death was the most common cause of abandonment in the RA group (66.7 vs. 30.2%) while access failure was the most common cause of abandonment in the GA group (69.8 vs. 33.3%). The most common cause of access failure in both groups was thrombosis, followed by poor flow.

Overall complication rates were comparable in both the groups (20.2 vs. 17.5%, P= 0.816), with common complications being stenosis, steal syndrome, and hematoma formation/bleeding. A trend of a higher rate of interventions was noted in the GA vs. RA group (18.2 vs. 12.8%). A higher rate of fistula salvage (80% vs. 61.1%) was noted in RA after an intervention. A significantly higher number of patients in RA developed anesthesia-related pulmonary complications as compared to GA (7.1 vs. 0.9). No significant difference in total length of hospital stay or high dependency unit (HDU) stay in both groups but a significantly higher rate of ICU stay was noted in RA as compared to the GA group (4.8 vs. 0%).

## Discussion

In this study, we compared the outcomes of BT AVF in RA vs. GA in terms of maturation, patency, and complications. RA success rates in our study are comparable to those reported in the literature [17]. A trend of higher maturation rates and higher six-month and 12-month patency rates was observed in patients undergoing procedures in RA. Excellent maturation rates are seen in both the exposure groups (92.1% vs. 97.6% in GA vs. RA, respectively) with very low primary failures, reflecting higher maturation rates than reported by Al-Jaishi AA et al. and Voorzat et al. for upper arm AVFs [6,18]. Al-Jaishi et al. has conducted a meta-analysis on the patency of arteriovenous fistulae, including both forearm and upper arm AVF [6] while Voorzat et al. has investigated patency outcomes as well as complications in both AVFs and arteriovenous grafts (AVGs) [18]. The reason for higher maturation in our study group may be because we have studied only BT AVF as compared to Al-Jaishi et al. and Voorzat et al. [6,18] who have studied BC AVF as well as BT AVF together and BT AVF is reported to have higher maturation rates than BC AVF [19]. One-year patency in our study is comparable to that reported by Al-Jaishi et al. and Voorzat et al. reported estimates [6,18]. Aitken et al. conducted a randomized controlled trial comparing RA vs. local anesthesia (LA) in patients undergoing RC AVF and BC AVFs and concluded improved patency at three months in AVFs performed under RA [20]. Recently, Jorgensen MS et al. have compared the patency of 238 AVFs and AVGs performed under RA vs. GA, and they also concluded decreased failure rates at two months, with RA compared to GA [15]. Both of the above-mentioned studies are limited by short-term follow-up of three and two months, respectively. Our findings are similar to Jorgensen MS et al. and Aitken E et al. [15,20] showing a trend toward higher patency rates in the RA group at six and 12 months. The RA group in our study had a high proportion of elderly, ASA IV, and IHD patients compared to GA, and this proportion is also higher as compared to reported literature [15,18,21]. Keeping in view the high proportion of elderly and morbid patients in RA, this trend of higher maturation and patency rates is an important finding and may result in statistically significant differences if both groups were comparable in terms of demographics and clinical characteristics. Jorgensen MS et al. [15] found death as the most common cause of abandonment of AVF in both the RA as well as GA groups, but in our study, death is the most common cause of abandonment in the RA group while access failure is the most common cause of abandonment in the GA group. This finding again indicates that RA was chosen for patients who were morbid having significantly higher comorbids and ASA IV.

Although Robert J et al. [22] found decreased complication rates in RA as compared to GA, in our study, similar to Jorgensen MS et al. [15] and Cole NM et al. [21], we found no difference between the two techniques in terms of AVF-related complication rates. Although none of the patients developed cardiac complications peri-operatively, contrary to our assumption, three (7.1%) patients in the RA group developed respiratory complications as compared to one (0.9%) patient in the GA group in the postoperative period. Two of the patients in the RA group, developing respiratory complications, needed ICU stay while no patient in the GA group needed ICU stay. This higher number of pulmonary complications and ICU stay in the RA group may be explained because of the high number of elderly and morbid patients in the RA group as compared to the younger and less morbid population of the GA group. No difference was observed in the rate of interventions to salvage the fistulae between the two groups but a trend of higher post-intervention salvage rate was seen in the RA group. Cole NM et al. [21] found decreased hospital stay with RA as compared to GA, but in our study, we found no difference in length of stay between the two groups.

This study will fill gaps in loco-regional data on outcomes of BVT AVF when performed under RA vs. GA with some limitations. This is a single-center, retrospective study that carries some inherent limitations, including loss of follow-ups, missing data on potential confounding factors, with the possibility of non-homogeneous definitions of outcomes recorded by different levels of health professionals over the course of the study. However, this limitation is applied to the whole sampled population.

## Conclusions

Regional anesthesia is a useful technique with potentially improved maturation and patency rates. Nevertheless, the assumed benefit of regional anesthesia in terms of anesthesia-related complications was not observed. A randomized, prospective, multicenter study with a larger number of patients with comparative demographics and clinical risks may overcome the limitations of this study.
